# Tamoxifen-elicited uterotrophy: cross-species and cross-ligand analysis of the gene expression program

**DOI:** 10.1186/1755-8794-2-19

**Published:** 2009-04-28

**Authors:** Joshua C Kwekel, Agnes L Forgacs, Lyle D Burgoon, Kurt J Williams, Timothy R Zacharewski

**Affiliations:** 1 Department of Biochemistry and Molecular Biology, Michigan State University, East Lansing, MI, USA; 2 Department of Pathobiology & Diagnostic Investigation, Michigan State University, East Lansing, MI, USA; 3 Center for Integrative Toxicology and National Food Safety and Toxicology Center, Michigan State University, East Lansing, MI, USA

## Abstract

**Background:**

Tamoxifen (TAM) is a well characterized breast cancer drug and selective estrogen receptor modulator (SERM) which also has been associated with a small increase in risk for uterine cancers. TAM's partial agonist activation of estrogen receptor has been characterized for specific gene promoters but not at the genomic level *in vivo*.Furthermore, reducing uncertainties associated with cross-species extrapolations of pharmaco- and toxicogenomic data remains a formidable challenge.

**Results:**

A comparative ligand and species analysis approach was conducted to systematically assess the physiological, morphological and uterine gene expression alterations elicited across time by TAM and ethynylestradiol (EE) in immature ovariectomized Sprague-Dawley rats and C57BL/6 mice. Differential gene expression was evaluated using custom cDNA microarrays, and the data was compared to identify conserved and divergent responses. 902 genes were differentially regulated in all four studies, 398 of which exhibit identical temporal expression patterns.

**Conclusion:**

Comparative analysis of EE and TAM differentially expressed gene lists suggest TAM regulates no unique uterine genes that are conserved in the rat and mouse. This demonstrates that the partial agonist activities of TAM extend to molecular targets in regulating only a subset of EE-responsive genes. Ligand-conserved, species-divergent expression of carbonic anhydrase 2 was observed in the microarray data and confirmed by real time PCR. The identification of comparable temporal phenotypic responses linked to related gene expression profiles demonstrates that systematic comparative genomic assessments can elucidate important conserved and divergent mechanisms in rodent estrogen signalling during uterine proliferation.

## Background

The estrogen receptor (ER) is a master transcriptional regulator involved in the proliferation and differentiation of many tissues, most notably the female reproductive tract. It functions as a ligand-dependent transcription factor with two activation functions (AF-1 and AF-2) which interact with cofactors (SRC-1, GRIP1, CBP/p300) to modulate transcription using different mechanisms [1,2]. The genomic activities of ER are mediated via direct DNA binding at estrogen response elements (EREs) or through indirect tethering mechanisms involving AP-1, Sp1, or Nf-κB [3]. It also has been shown to use non-genomic mechanisms via membrane associated ERs which activate various protein kinase cascades [4]. Furthermore, two distinct ER isoforms exist which have divergent functionality as well as tissue- and cell type-specific expression. For example, whereas mammary tissue expresses both ERα and ERβ [5] at comparable levels in different cells, the uterus predominantly expresses ERα.

Tamoxifen (TAM) is a well characterized breast cancer drug and prophylactic that is a selective estrogen receptor modulator (SERM). SERMs are structurally diverse compounds that bind the ER and elicit ligand- and tissue-specific effects [6,7], such as inhibiting the proliferative effects of estrogen in ER-positive breast cancers, while maintaining partial agonistic activity in other tissues [8]. SERM binding causes a unique conformation in the ER ligand binding domain which alters the apposition of ER helix 12 and function of AF-2 relative to 17β-estradiol (E2), while still allowing for the ligand-independent functionality of AF-1 [9].

TAM-induced ER-mediated gene expression has been characterized on a promoter/gene specific basis [10,11]. However, the effect of TAM on global uterine gene expression has not been comprehensively examined. Whether or not the modulation of helix 12/AF-2 by TAM results in merely decreased efficacy or specificity for typical ER gene targets or potentiates unique cofactor interactions and thus novel genomic targets has not been fully examined. Therefore, elucidating the genomic targets of TAM is important to the understanding of SERM-ER proliferative activities, especially due to its association with uterine cancer following continuous treatment [12], which is currently understood to be a function of its partial agonist activity.

The classic rodent uterotrophic assay to assess the estrogenic potential of a chemical examines both the physiological and histomorphological endpoints in the uterus [13]. Uterine wet weight, water imbibition and luminal epithelial cell height induction are typically evaluated after three consecutive daily treatments [14]. This assay can be extended by including temporal gene expression analysis, which can be anchored to these more apical endpoints in order to provide a more comprehensive assessment of the molecular and physiological effects of treatment [15,16].

Furthermore, this enriched assessment can also be used to evaluate the ability of surrogate models to accurately predict human responses to drugs, industrial chemicals, natural products and environmental contaminants. In the case of tamoxifen metabolism rats have been shown to exhibit a metabolic profile more similar to humans than mice [17,18]. Thus elucidating the dose-responsive and temporal effects of tamoxifen on gene expression will inform the relative importance of this divergence in rodent metabolic profile. Furthermore, elucidating species-specific differences in both rats and mice for either gene function or regulation is also an important factor for reducing uncertainties associated with cross-species extrapolation of data in risk assessment. Therefore, a cross-species comparative method was employed to examine temporal gene expression in rodents during TAM and ethynylestradiol (EE) induced uterotrophy in order to better understand the early molecular events associated with tamoxifen-related uterine cancers in humans.

## Results

### Uterine Wet Weight (UWW) and Water Content

**Figure 1:**
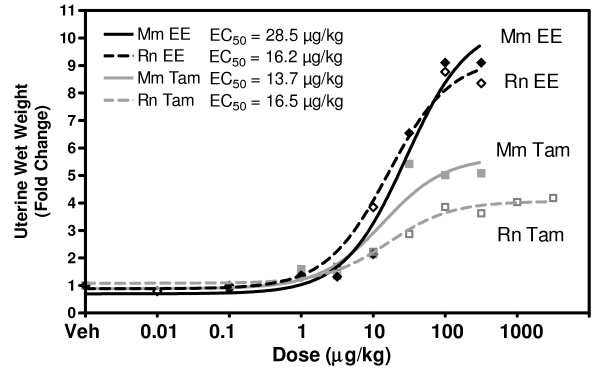
**Dose Response Uterine Wet Weights (UWW)**. UWW was measured across several EE and TAM doses in the mouse and rat. A plot of the fold change increase in wet weight is plotted. A dose response curve was fit to the data (GraphPad 4.0) to estimate EC_50 _values. 100 μg/kg b.w. approximates the maximum response in all four cases and was used in subsequent time course studies. ED_50 _values were comparable between ligands in the rat while exhibiting only a two fold difference in the mouse, indicating conservation of sensitivity to EE and TAM.

Increases in UWW were used to evaluate the responsiveness to EE and TAM. Dose response studies were performed using 0.01, 0.1, 1, 10, 100, 300 μg/kg b.w. EE, and 3, 10, 30, 100, 300, 1000, 3000 μg/kg b.w. TAM. In each case, 100 μg/kg approached the maximum uterotrophic response and this dose was subsequently used for the time course studies (Figure 1). Whereas TAM was equipotent to EE in eliciting uterotrophy in C57BL/6 mice and Sprague-Dawley rats, it was 43% less efficacious in both species eliciting only 4- and 5-fold induction (in the rat and mouse, respectively) compared to the ~9-fold increase induced by EE. In the time course studies, 100 μg/kg of EE or TAM was orally administered once daily for three consecutive days. UWW and water content changes were measured at each time point [see Additional File 1 and 2]. EE induced UWW increases only at 24 and 72 hrs in the mouse, while in the rat the classic water imbibition response occurred between 4 and 12 hrs followed by maximum induction at 72 hrs. TAM induction of water imbibition was delayed approximately 8 hrs in the rat and subdued in both (45% and 65% of EE in rat and mouse, respectively). The increases in uterine water content suggest that early increases in UWW are due at least in part to this water imbibition. As in the wet weight, the changes in water content after TAM treatment temporally lagged behind EE and were notably less efficacious. Therefore, the large difference in wet weight between EE and TAM at 72 hrs is possibly due to early differences in gene expression responsible for water imbibition.

### Histomorphology

**Figure 2:**
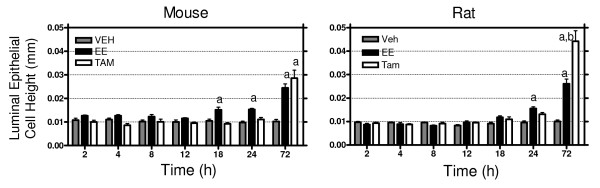
**Temporal Changes in Luminal Epithelial Height (LEH)**. LEH was quantified for each treatment group and compared to VEH controls. LEH induction was temporally delayed in response to TAM treatment in both species. Mouse induction was comparable between ligands while TAM-induced LEH was significantly (p < 0.05) greater than EE in rats.

Induction of luminal epithelial height (LEH) is a classic estrogen response [19,20] in the rodent uterus. No increases in LEH were observed in any group between 2 and 12 hrs (Figure 2). However, EE induced LEH as early as 18 hrs in the mouse and 24 hrs in the rat. While EE and TAM produced comparable levels of LEH induction (~2.6-fold) in the mouse at 72 hrs, TAM treatment in the rat elicited a significantly greater LEH increase (p < 0.05, 4.4-fold for TAM and 2.6-fold for EE). EE induced moderate to marked stromal edema beginning as early as 4 and 8 hrs while TAM induced severe stromal and myometrial edema at 12 hrs. Proliferative indices (number of mitotic bodies) were noted in EE treated uteri at 18 and 24 hrs but not detected in TAM treated samples. Moderate to severe hyperplasia and hypertrophy in the stroma and epithelium were present at 72 hrs in all studies. There was mild apoptotic cell death in the epithelium in TAM studies at 72 hrs while mild to marked apoptosis was noted in EE samples. The species difference in uterine architecture (luminal invaginations seen in the mouse but not rat) previously observed [21] at 72 hrs was not as pronounced in TAM treated samples.

### Comparative Species and Ligand Analysis of Gene Expression

**Figure 3:**
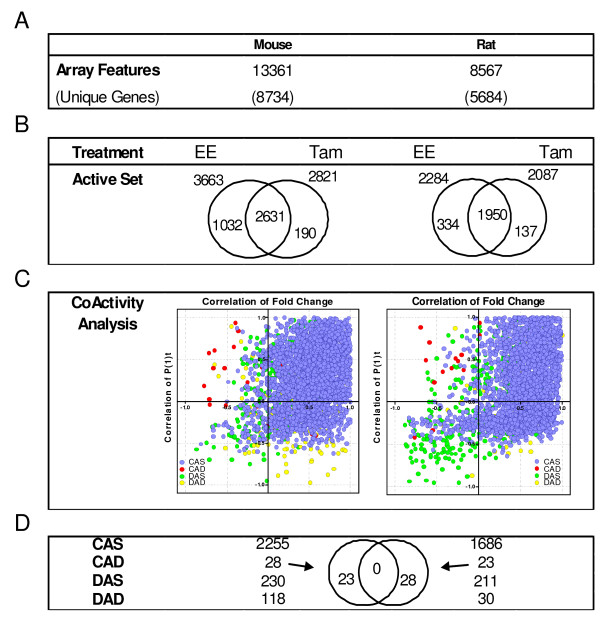
**Comparative Analysis of Species-Conserved, Ligand-Specific Gene Expression**. (A) cDNA microarrays were used containing 13,361 mouse clones representing 8,734 unique genes and 8,507 rat clones representing 5,684 unique genes. (B) Differentially expressed genes regulated by each ligand were identified using relaxed criteria to minimize the likelihood of false-negatives that marginally failed to meet the selection criteria. Differentially expressed genes elicited by both EE and TAM were analyzed for similarity in their temporal profiles by comparative analysis. Genes were designated as either CoActive-Similar direction (CAS), CoActive-Divergent direction (CAD), Displaced Active Similar direction (DAS), or Displaced Active Divergent direction (DAD) based on the relationship between the time and direction of differential regulation, and the significance (P1(*t*)) of the expression profile relative to the VEH control. (C) The comparative results were plotted on a coordinate correlation graph. A majority of genes show positive correlation between ligands for both fold change and P1(*t*) value. (D) Cross species analysis of ligand-divergent (CAD) expression profiles indicate no conservation.

Global gene expression changes with respect to VEH controls were measured and compared across time for MmEE, MmTAM, RnEE, and RnTAM treatments. Pair-wise comparisons between compounds per species and between species per compound were made to investigate conserved responses. A two-tiered, bipartite (P1(*t*) and fold change) approach was used to screen for conserved differentially expressed genes (Figure 3).

In the mouse, 3,663 and 2,821 genes were differentially regulated by EE and TAM respectively, with 2,631 common differentially expressed genes across all time points (Figure 3B). These 2,631 common, differentially expressed genes were then further examined to assess the similarity of their EE- and TAM-elicited temporal expression profiles by comparing their expression profiles in order to assess similarity between the treatments (Figure 3C). This comparison is comparable to a correlation analysis in assessing the similarity of statistically significant differential gene expression relative to a control elicited by EE and TAM. Each differentially expressed gene was designated as either coactive-similar direction (CAS), coactive-divergent direction (CAD), displaced active-similar direction (DAS), or displaced active-divergent direction (DAD) based on the relationship between the time and direction of differential regulation, and the significance (P1(*t*)) of the expression profile relative to the VEH control. For example, Igf1 is designated as CAS in the rat because it was up regulated in a similar temporal pattern by both EE and TAM between 12–24 hrs, while Junb was designated DAS because although it was up regulated by both compounds, EE induction was at 4 hrs but TAM treatment temporally shifted the induction to 12 hrs. These designations assist in developing gene lists and provide a preliminary assessment of the similarity in gene expression profile on a qualitative level that takes into account the statistical difference from VEH which typically is not considered in correlation analyses. Using this approach, > 85% of EE and TAM induced genes were designated as CAS (2255 genes). Only a few were categorized as CAD or "divergently regulated" (28 genes, 1% of the overlap).

Similarly in the rat, 2,284 and 2,087 genes were differentially expressed by EE and TAM, respectively, resulting in 1,950 commonly regulated genes (Figure 3B). In both species, roughly 93% of TAM-regulated genes overlapped with EE. Of the 1,950 genes differentially expressed by both EE and TAM in the rat, 86% (1,686 genes) were designated as CAS while only 23 were CAD. Comparison of the 28 mouse CAD genes to the 23 rat CAD genes found no overlapping orthologs (Figure 3D), suggesting that there are no conserved differentially regulated genes for which EE and TAM elicit divergent uterine responses in the rat and mouse.

### Comparison of Orthologous Gene Expression

**Figure 4:**
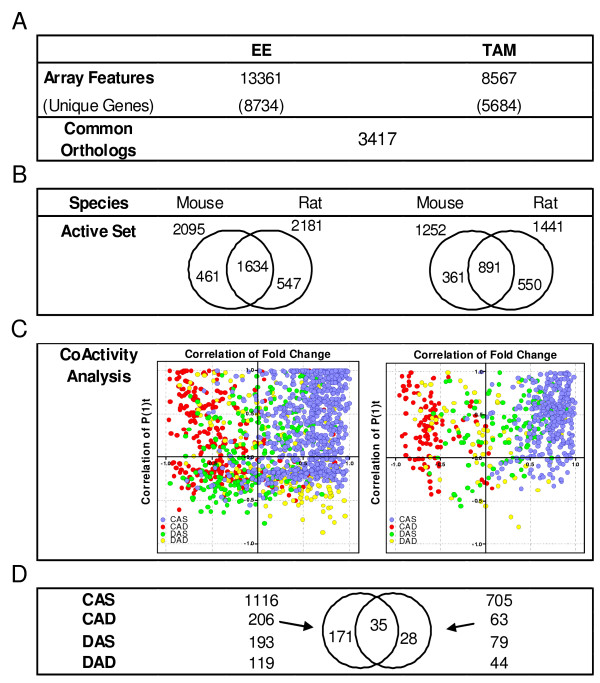
**Comparative Analysis of Ligand-Conserved, Species-Specific Gene Expression**. (A) cDNA microarrays were used containing 13,361 mouse clones representing 8,734 unique genes and 8,507 rat clones representing 5,684 unique genes. There were 3,417 orthologous genes represented on the mouse and rat cDNA microarrays as determined by HomoloGene. (B) Differentially expressed genes were assessed for similar expression patterns using relaxed criteria (|fold change| > 1.3; P1(*t*) > 0.99) to minimize the likelihood of false-negatives that marginally failed to meet the selection criteria. (C) Common differentially expressed orthologous genes were examined for comparable expression patterns, and designated according to the coactivity categories (CAS, CAD, DAS, DAD) as described in Figure 3. The results were plotted on a coordinate correlation graph. A high proportion of differentially expressed orthologs exhibited a positive correlation when considering both fold-change and P1(*t*). (D) Comparisons of species-divergent (CAD) expression profiles identified 35 genes that were differentially expressed in the mouse and the rat but exhibited putative divergent regulation elicited by EE and TAM.

A comparable approach was used to examine the cross-species differential gene expression effects of EE and TAM on orthologous rat and mouse genes. 3,417 unique orthologous genes were represented on the rat and mouse array platforms as determined by NCBI's HomoloGene database (Figure 4A). In response to EE treatment, 2,095 and 2,181 of the 3,417 orthologous genes were differentially expressed in the mouse and rat, respectively, with 1,634 orthologs differentially expressed in both species (~75%) (Figure 4B). These 1,634 differentially expressed orthologs were analyzed for coactivity of which 1,116 genes (68%) were designated as CAS and 206 CAD. Similarly, 1,252 mouse and 1,441 rat genes were differentially expressed following TAM treatment with 891 orthologs differentially expressed in both species. 705 (79%) of these were designated as CAS genes while 63 genes were designated CAD. A comparison of the EE and TAM CAD orthologs identified 35 genes that are divergently regulated between the mouse and rat in response to both EE and TAM. Of these 35 genes, 9 were represented by only a single feature on the microarray for both species, 12 were represented by 2 or more features in at least one species that had poor internal correlation, and 14 were represented by 2 or more features in at least one species that were internally consistent. Thus, a strength of evidence approach was taken for pursuing genes exhibiting putative divergent regulation with quantitative real time PCR.

### QRT-PCR Verification of Microarray Data

The expression profiles of 25 genes (including known estrogen responsive genes: Fos, C3, Calb3, Igf1, Sult1a1, Aqp5, and Vegf) accounting for the spectrum of temporal patterns and functional categories were verified by QRT-PCR in either species or compound. In general there was a good correlation between the QRT-PCR and microarray results (data not shown).

**Figure 5:**
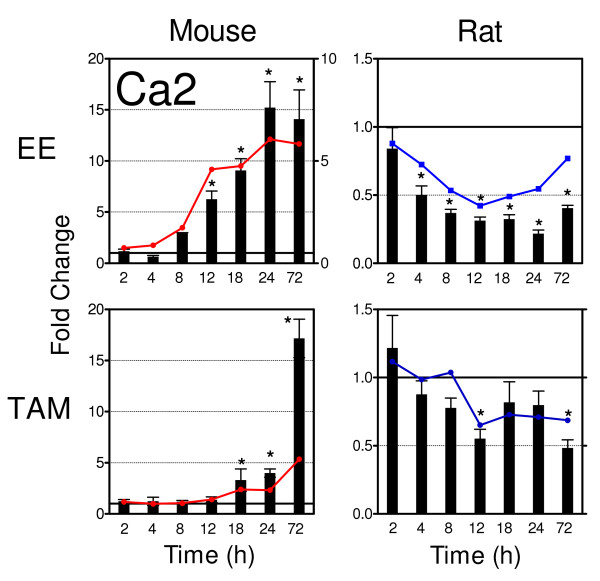
**Quantitative Real Time PCR Confirmation of Species-Specific ER Regulation of Ca2**. The temporal gene expression of carbonic anhydrase 2 (designated Car2 in the mouse and Ca2 in the rat and human, hereafter referred to as Ca2 to represent all orthologs) was further validated by species-conserved and -unique primer sets. The QRT-PCR results confirm the microarray results indicating that Ca2 is differentially regulated in the mouse when compared to the rat. This differential regulation was elicited by both EE and TAM. Mouse Ca2 expression is significantly (p < 0.05) induced while rat expression is significantly (p < 0.05) repressed (n = 5, Tukey's HSD post hoc test), at multiple time points as indicated by the asterisk (*).

Fourteen orthologs that exhibited putative divergent regulation in the mouse compared to the rat following EE and TAM treatment were further investigated. However, QRT-PCR indicated that these genes exhibited either a CAS relationship between species or were inconclusive, except for carbonic anhydrase 2 (designated Car2 in the mouse and Ca2 in the rat and human, hereafter referred to as Ca2 to represent all orthologs). QRT-PCR analysis of Ca2 confirmed the microarray data (correlation coefficient r = 0.95, 0.98, 0.82, and 0.78 for MmEE, MmTAM, RnEE and RnTAM, respectively; Figure 5). These results were confirmed by two different sets of primers, with one set querying species specific regions of the mouse and rat mRNA, and the second set designed to amplify the same region in both species using the same primers.

### Four-Way Venn Analysis

**Figure 6:**
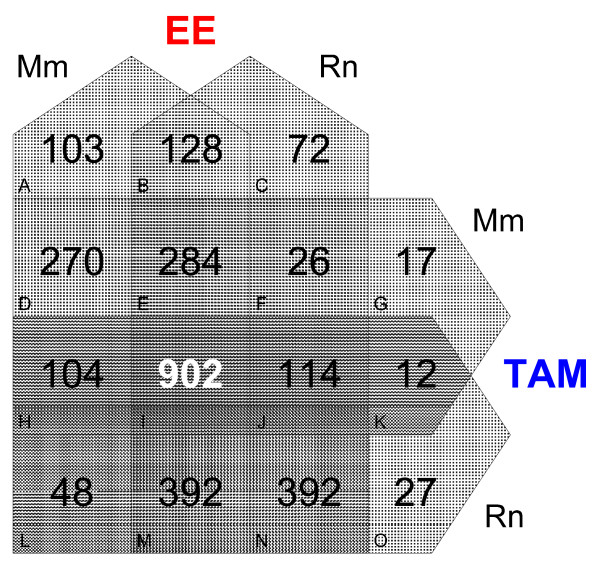
**Four Way Venn Analysis of Active Genes**. Each data set was converted into ortholog space and processed for activity using relaxed criteria prior to analysis using the 4-way Venn Diagram Generator . The Venn set was subsequently filtered for genes which met the initial, high-stringency criteria to ensure robust comparisons. Gene lists for each Venn set are available [see Additional File 7].

Following pair-wise comparisons by chemical and species, an integrated comparison of all four data sets was performed where differentially expressed orthologous genes (NCBI, HomoloGene) for each data set were entered into the 4-way Venn Diagram Generator. The number and identity of unique genes populating the overlaps of each data set was determined for common orthologs (Figure 6). The comparison of all four data sets resulted in 902 genes being commonly regulated at any time point (Venn subset I). Of the 902 overlapping genes, 398 exhibited a similar temporal profile (CAS) across all four data sets, suggesting comparable modes of regulation which we refer to as "orthologous expression". These genes represented functional categories associated with cellular proliferation and differentiation; hallmarks of estrogen induced uterotrophy. Selected conserved examples exhibiting orthologous expression in terms of either fold-change alone (Fos, C3, Igf1, Ca3, Sepp1) or large increase in copy number, regardless of fold change (Cd24a, Slc25a5, Krt13, Dcn, Itm2b) are provided in Table 1.

**Table 1:** Species and Compound Conserved Genes

			**Maximum Fold Change**				
**Functional Category**	**Gene Symbol**	**Entrez Gene ID (Mm)**	**MmEE**	**RnEE**	**MmTAM**	**RnTAM**	**Time**^a^
**Energetics**	Aldoa	11674	**1.36**	**2.04**	**1.34**	**1.75**	Sustained
	Atp5b	11947	**1.70**	**1.85**	**1.67**	**1.69**	Sustained
	Atp5g1	11951	**3.77**	**2.57**	**2.86**	**1.88**	Sustained
	Ckb	12709	**2.72**	**2.92**	**1.42**	**2.04**	Mid
	Cox7b	20463	0.28	0.49	0.75	0.57	Sustained
	Cycs	13063	**4.69**	**3.72**	**4.22**	**3.11**	Sustained
	Ldha	16828	**2.60**	**2.46**	**2.66**	**2.20**	Sustained
	Pgk1	18655	**3.77**	**3.25**	**2.46**	**2.93**	Sustained
	Slc25a4	11739	**2.02**	**1.94**	**1.53**	**1.94**	Mid
	Slc25a5	11740	**5.32**	**3.17**	**4.31**	**3.09**	Sustained
	Txnl2	30926	**1.96**	**2.35**	**1.45**	**1.90**	Mid/Sustained

**Chaperone & Protein Folding**	Cct7	12468	**2.76**	**2.71**	**2.18**	**2.08**	Sustained
	Hsp90aa	15519	**2.65**	**1.47**	**3.21**	**2.69**	Mid
	Hspa5	14828	**4.44**	**2.35**	**6.72**	**2.32**	Sustained
	Hspd1	15510	**3.76**	**2.97**	**4.06**	**2.52**	Mid
	Hspe1	15528	**3.64**	**3.67**	**3.43**	**3.08**	Mid

**Cell Structure**	Dynll1	56455	**2.33**	**2.26**	**1.67**	**1.73**	Sustained
	Krt13	16663	**2.36**	**3.48**	**2.79**	**3.68**	Sustained
	Krt8	16691	**1.78**	**2.95**	**1.30**	**3.10**	Sustained
	Serpinh1	12406	**3.71**	**2.80**	**3.51**	**2.05**	Mid
	Tubb2c	227613	**1.35**	**3.07**	**3.03**	**2.68**	Mid

**Cell Signaling**	App	11820	0.40	0.39	0.48	0.51	Sustained
	Cd24a	12484	**2.23**	**4.00**	**5.00**	**3.74**	Late
	Dcn	13179	0.35	0.38	0.50	0.49	Sustained
	Igf1	16000	**4.50**	**7.16**	**4.40**	**5.92**	Sustained
	Sepp1	20363	0.26	0.25	0.19	0.31	Mid

**Transcription & NA Processing**	Atf4	11911	**4.42**	**3.27**	**2.96**	**2.00**	Sustained
	Cnot4	53621	**3.16**	**6.33**	**2.88**	**5.91**	Sustained
	Fos	14281	**6.38**	**40.25**	**4.08**	**7.86**	Early
	Hnrpab	15384	**3.31**	**3.17**	**2.28**	**2.18**	Sustained
	Hnrpu	51810	**2.22**	**1.82**	**1.71**	**1.49**	Sustained

**Translation & Protein Turnover**	Eif1	20918	**1.57**	**2.37**	**1.41**	**1.71**	Mid
	Eif2s2	67204	**3.50**	**1.87**	**1.82**	**2.38**	Sustained
	Psma5	26442	**1.64**	**1.99**	**1.42**	**1.70**	Mid
	Psmb5	19173	**3.11**	**2.31**	**2.33**	**1.90**	Mid

**Uncategorized or Other**	Armet	315989	**9.77**	**3.67**	**2.21**	**3.11**	Sustained
	C3	12266	**3.62**	**9.27**	**5.36**	**4.85**	Late
	Car3	12350	0.06	0.11	0.12	0.31	Late
	Cst3	13010	0.72	0.34	0.61	0.35	Sustained
	Hba-a1	15122	0.25	0.40	0.41	0.49	Late
	Itm2b	16432	0.52	0.21	0.48	0.35	Mid
	Ly6e	17069	**1.90**	**2.49**	**1.96**	**1.38**	Early/Late
	Mgp	17313	0.22	0.40	0.38	0.51	Sustained
	Ran	19384	**3.23**	**4.25**	**3.68**	**3.19**	Mid
	Txn1	22166	**1.51**	**2.10**	**1.64**	**1.72**	Mid

a Early = 2, 4 or 8 hrs, Mid = 8, 12, 18 or 24 hrs, Late = 72 hrs, Sustained = 3 or more consecutive timepoints.

Of interest are the 128 and 12 genes populating subsets B and K, respectively. Subset B represents putative species-conserved EE-specific responses, of which, less than 60% exhibited similar direction of regulation (CAS or DAS designation) by TAM. Most of these genes were regulated by TAM but did not meet the fold change or P1(*t*) selection criteria, suggesting a potency issue rather than unique regulation. For example S100a8, a calcium binding protein, was induced 1.5-fold (Mm) and 3.5-fold (Rn) by EE while TAM only induced a 1.3-fold change in the mouse that did not meet the statistical cutoff while in the rat the statistical cutoff was achieved but induction was only 1.2-fold. Likewise, further analysis of the 12 species-conserved TAM-specific responses revealed that only Myh6 (myosin, heavy chain 6), a component of muscle fibre that is likely expressed in the myometrium, exhibited an overlap in temporal expression between species. However, comparable expression is only observed at 72 hrs where secondary and tertiary effects are likely to overlap. In addition, there is insufficient data to exclude the possibility that Myh6 is regulated by TAM via an ER-independent mechanism.

Collectively, these results suggest there are no species-conserved, TAM-specific or -divergent genes which are regulated in a separate manner when compared to EE in the rodent uterus. Approximately 400 genes exhibited highly conserved and correlated expression across all four data sets, defining a robust gene set that is predictable and integral to ER-mediated uterotrophy in the rodent. However, species-specific gene expression profiles are difficult to assess and confirm given the complex orthologous relationships between the species specific array probe sets. Many of the putative differences suggested may be attributed to differing probe sensitivities or representations within respective genes. The lone identified exception is the species-divergent regulation of Ca2 that is conserved in response to both ER ligands and confirmed by homologous and species-specific QRT-PCR primer pairs.

## Discussion

The uterotrophic response has been used as an acute assay to assess the estrogenicity of a compound using both physiological and cytoarchitectural endpoints. Our extended design incorporates early monitoring of global gene expression (2–24 hrs) to capture the ER-mediated effects as well as subsequent secondary and tertiary responses. Early gene expression was also examined for conserved responses across ligand and species. The highly parallel nature of the EE and TAM study designs and subsequent analyses facilitated more robust comparisons, thus increasing confidence in the identification of conserved uterine responses between species and ligands.

EE and TAM doses were selected based on UWW dose response studies which identified 100 μg/kg as the most efficacious dose for each compound. Although differing pharmacokinetics may account for discrepancies between rodents and humans, 100 μg/kg TAM is below the pharmacological dose of Nolvadex^® ^(tamoxifen citrate, 20–40 mg/day) prescribed to women (300–400 μg/kg), assuming an average weight of 70 kg. Moreover, the ovariectomized model provides increased sensitivity to estrogen action in the immature rodent and thus allows for a more comprehensive assessment of the partial-agonistic activity of TAM in the uterus.

The physiological effects were comparable between species and ligand with EE eliciting the classic uterotrophic responses while TAM showed decreased efficacy with only partial agonist activity, as previously reported [22]. However, the more pronounced induction of luminal epithelial cell height in the rat compared to the mouse was unexpected. Previous rat studies have not observed increased efficacy of TAM in inducing luminal epithelial cell height relative to estrogen [23,24], which may due to differences in animal age, ovariectomy status or other study design issues. However, a comparable increase in TAM LEH induction in rats relative to estrogen was previously reported as not statistically different [25]. Furthermore, the uterotrophic effects of EE and TAM in ovariectomized Cynomolgus Macaque monkeys [26] showed a noticeable increase in TAM induced LEH relative to EE that was not statistically significant. The similarity in LEH response to higher order mammals/primates suggests that the rat may be a better model of the human response, but further study is required.

The partial agonist effects of TAM with regard to its ER-complex conformation and differential interactions at specific canonical response elements (including EREs and AP-1 sites) as well as gene specific promoters (Vitellogenin, Complement component 3, prolactin) have been well studied. However, the global gene targets of TAM-ER complex have not been comprehensively elucidated in the rodent uterus. Although direct primary ligand-ER effects can not be deduced from expression profiling alone, our comparative approach provides evidence of several conserved responses elicited by two structurally diverse ligands. While EE is absorbed and attains peak plasma concentration before TAM does [27,28], the difference is only a matter of hours and the more likely limiting step is the hydroxylation of TAM to 4-OH-tamoxifen or endoxifen, which could account for the 10–12 h delay observed in gene expression response between EE and TAM. However, as primary gene responses give way to secondary responses, gene expression cascades are sequentially propagated across time. If the two ligand-receptor complexes elicit different behaviours at the primary response genes, it is likely that subsequent differences in secondary and tertiary responses would also be propagated over time. However, this is not the case as EE and TAM elicited comparable expression profiles that are maintained throughout the time course in both species. This suggests that TAM elicits parallel uterine gene expression behavior when compared EE, despite the temporal shift of expression due to pharmacokinetic differences [29].

An inclusive, multi-step approach to identifying differentially expressed genes was used to ensure conservation of gene expression between ligand or species could not be attributed to strict cut offs applied during screening. Comparison of gene expression profiles revealed highly similar sets of genes between species and compounds (Figure 3 and 4). When profiles were aligned and compared on a quantitative scale, TAM typically elicited a lower fold change when compared to EE, consistent with its partial agonist activity. However, four notable species-conserved orthologs (Hsp90aa1, Slc9a3r1, Acsbg1, Col5a1) were identified where TAM induction exceeded that of EE. For example, Slc9a3r1, a cytoskeletal-membrane protein binding-protein that functions to maintain epithelial cell structure and polarity [30], was induced 4.1-fold (Rn) and 3.6-fold (Mm) by TAM but only 2.7-fold (Rn) and 3-fold (Mm) by EE, respectively. Few genes exhibited unique differential gene expression suggesting that TAM elicits uterotrophic affects through the same target genes affected by EE (Figure 6). This is significant, as little information is known about how conformational changes in the SERM-ER complex affect the number or types of uterine genes modulated after treatment.

A large number of regulated genes were differentially expressed across all four data sets, of which, 398 exhibited comparable patterns of expression [see Additional Files 3, 4, 5, and 6], and likely represent conserved ER-mediated uterine responses. They were associated with several functional categories including cellular energetics, chaperone and protein folding, cell structure, cell signaling, transcription and RNA processing, and translation and protein turnover (Table 1). Although most genes were up regulated, those associated with the cell cycle and mitogenic activity were down regulated, consistent with an overall proliferative response [31,32]. Several of these conserved early (e.g., Fos and Vegf), mid (e.g., Ccnd1) and late (e.g., C3) responders responses are known ER targets regulated via estrogen response elements (EREs), AP-1, and Sp1 sites. The temporal differences in expression (between 2 and 72 hrs after treatment) of genes known to be ER regulated affirms the bi- or multi-phasic activities of activated ER during uterotrophy [33]. Although the TAM profiles exhibited a temporal shift for early regulated genes (Fos and Vegf), genes differentially expressed at 18, 24 and 72 hrs (Ccnd1, Tk1) exhibited little temporal shift. This suggests a pharmacokinetic difference which is not applied equally to all responses or is masked by the sustained nature of mid to late gene responses.

TAM perturbs calcium homeostasis causing intracellular influx of Ca^2+ ^ions which is related to cytotoxic, thrombotic and systemic effects [34,35]. This is believed to be ER independent, and is suggestive of alternative mechanisms activated only by TAM. However, no differential gene expression was observed regarding genes involved in calcium homeostasis, including Ca^2+ ^transporters Atp2a1, -2a2, -2b1, and -2b2, calcium transporters that are targets of the calcineurin/Crz1 signaling pathway which regulates intracellular Ca^2+ ^homeostasis. Moreover, the increased LEH responsiveness in rat is not explained by our current data, although this may be due to differences in genome coverage represented on our mouse and rat cDNA microarrays. It is also likely that there are significant species-specific post-transcriptional differences that are not assessed using gene expression approaches.

## Conclusion

In summary, our parallel study design afforded a robust comparative analysis of EE and TAM elicited responses in the rat and mouse uterus. TAM induced UWW and LEH in a manner consistent with its partial-agonist activity at a dose equipotent to EE. This suggests that EE and TAM elicit comparable uterine gene expression profiles despite conformation differences in the liganded ER complexes, and that the transcriptional activity via AF-1, which is activated by both EE and TAM, is sufficient to elicit uterine differential gene expression. This is consistent with mice studies with targeted disruption of the ER DNA binding domain [36], which did not exhibit acute uterotrophy but still elicited epithelial proliferation and increased LEH. It also supports the sufficiency of the AF-1 domain alone (via TAM binding) to mediate uterotrophy. Although differences in LEH induction were observed between EE and TAM, collectively our data suggests that TAM does not elicit a unique uterine gene expression profile when compared to EE. However, more comprehensive studies are warranted that would examine the differential expression of all orthologous rat and mouse genes.

## Methods

### Husbandry

Experimental designs and methods for the rat tamoxifen data parallel previously published rat and mouse EE and mouse TAM studies [21,37]. Briefly, female Sprague-Dawley rats and C57BL/6 mice, ovariectomized on PND 20 and all within 10% of the average body weight were obtained from Charles River Laboratories (Raleigh, NC) on day 25. Animals were housed in polycarbonate cages containing cellulose fibre chip bedding (Aspen Chip Laboratory Bedding, Northeastern Products, Warrensberg, NY) and maintained at 40–60% humidity and 23°C in a room with a 12 hrs dark/light cycle (7 am–7 pm). Animals were allowed free access to de-ionized water and Harlan Teklad 22/5 Rodent Diet 8640 (Madison, WI), and acclimatized for 4 days prior to dosing.

### Treatments

In dose response studies, animals received three consecutive, daily oral treatments of EE (0.01 to 300 μg/kg) or TAM (3 to 3000 μg/kg b.w.). Animals were sacrificed 72 hrs after the initial dose. In time course studies animals were treated once or once daily for three consecutive days via oral gavage with 100 μg/kg b.w. EE or TAM in 0.1 mL of sesame oil vehicle (Sigma Chemical, St Louis, MO), (EE time course treatment in mouse, MmEE; EE time course treatment in rat, RnEE; EE in mouse, MmEE; TAM in mouse, MmTAM;). This oral dose was empirically derived and chosen because it elicited a maximal uterotrophic response in both species while showing no acute toxic effects. Animals receiving a single dose were sacrificed 2, 4, 8, 12, 18, or 24 hrs after treatment. Animals receiving three consecutive, daily doses were sacrificed 72 hrs post initial dose. An equal number of time-matched vehicle control (VEH) animals were treated in the same manner, (n = 8 for MmEE, n = 5 for all others). Doses of EE and TAM were calculated based on average weights of each treatment and VEH group prior to dosing. All procedures were performed with the approval of the Michigan State University All-University Committee on Animal Use and Care.

### Necropsy

Animals were sacrificed by cervical dislocation and animal body weights were recorded. The uterine body was dissected at the border of the cervix and whole uteri were harvested and stripped of extraneous connective tissue and fat. Whole uterine weights were recorded before (wet) and after (blotted) being blotted under pressure with absorbent tissues and were subsequently snap-frozen in liquid nitrogen and stored at -80°C. Weight due to water was calculated as the difference between the wet and blotted weights. A small (~5 mm) section of the right, distal uterine horn was placed in 10% neutral buffered formalin (NBF; VWR, West Chester, PA) and stored at RT for at least 24 hrs prior to further processing. Statistical analysis of wet weight and water content were conducted using a two-way ANOVA with a Tukey's Honestly Significant Difference post hoc test, p < 0.05 (SAS 9.1, SAS Institute Inc. Cary, NC).

### RNA Isolation

Total RNA was isolated from whole uteri (~20 mg/rat, ~3 mg/mouse) using Trizol^® ^Reagent (Invitrogen, Carlsbad, CA). Uteri were removed from -80°C storage and immediately homogenized in 1 mL Trizol^® ^Reagent using a Mixer Mill 300 tissue homogenizer (Retsch, Germany). Total RNA was isolated according to manufacturer's protocol and resuspended in The RNA Storage Solution (Ambion, Austin, TX). Concentration was calculated by spectrophotometric methods (A_260_) and purity assessed by the A_260_:A_280 _ratio and by visual inspection of 1 μg on a denaturing gel.

### Histological Processing

NBF-fixed uterine sections were routinely processed and embedded in paraffin according to standard histological techniques. Five μm cross-sections were mounted on glass slides and stained with hematoxylin and eosin. All embedding, mounting and staining of tissues were performed at the Histology/Immunohistochemistry Laboratory, located in the Department of Physiology, of Michigan State University.

### Histopathological and Morphometric Assessment

Histological slides were scored according to standardized National Toxicology Program (NTP) pathology codes. Morphometric analyses were performed on cross sections for each animal using image analysis software (Scion Image, Scion Corporation, Frederick, MD) and standard morphometric techniques. Briefly, the contour length of basal lamina underlying the luminal epithelium (LE) and corresponding areas of LE was quantified for multiple, representative sectors of each section. Statistical analyses on all morphometry data were performed using a two-way ANOVA with a Tukey's Honestly Significant Difference post hoc test, n = 5, p < 0.05 (SAS 9.1, SAS Institute Inc.).

### Microarray Platform

Rat cDNA arrays were produced in-house using a LION Bioscience's Rat cDNA library (LION Bioscience, Heidelberg Germany) consisting of 8,567 clones representing 5,684 unique genes (Unigene Build #48). Clones were selected based on their level of annotation as well as sequence similarity to well annotated human and mouse genes. Detailed protocols for microarray construction, labeling of the cDNA probe, sample hybridization and slide washing can be found at . Briefly, PCR amplified DNA was robotically arrayed onto epoxy coated glass slides (SCHOTT Louisville, KY), using an Omnigrid arrayer (GeneMachines, San Carlos, CA) equipped with 32 (8 × 4) or 48 (12 × 4) Chipmaker 2 pins (Telechem, Atlanta, GA) for the rat and mouse arrays, respectively, at the **Genomics Technology Support Facility at Michigan State University **.

### Array Experimental Design

Temporal changes in gene expression were assessed using an independent reference design in which samples from EE and TAM treated animals were co-hybridized with VEH. Comparisons were performed on 3 biological replicates × 2 independent labelings of each sample (incorporating a dye swap) for each time point. Total RNA (15 μg) was reverse transcribed in the presence of Cy3- or Cy5-labeled dUTP (Amersham, Piscataway, NJ) to create fluor-labeled cDNA, which was purified using QIAquick PCR purification kit (Qiagen, Valencia, CA). In contrast, note that mouse array experiments used a 3DNA Array 900 Expression Array Detection Kit (Genisphere, Hatsfield, PA) using 1 μg of total RNA, according to manufacturer's specifications, for probe labeling. Cy3- and Cy5-labeled samples were mixed, vacuum concentrated (~1–2 μl) and resuspended in 32 μl of hybridization buffer (40% formamide, 4× SSC, 1% SDS) with 20 μg polydA and 20 μg of mouse COT-1 DNA (Invitrogen) as a competitor. This probe mixture was heated at 95°C for 2 min and was then hybridized to the array under a 22 × 40 mm lifterslip (Erie Scientific Company, Portsmouth, NH) in a light protected and humidified hybridization chamber (Corning Inc., Corning, NY). Samples were hybridized for 18–24 h at 42°C in a water bath. Slides were then washed, dried by centrifugation and scanned at 635 (Cy5) and 532 nm (Cy3) on a GenePix 4000B microarray scanner (Molecular Devices, Union City, CA). Images were analyzed for feature and background intensities using GenePix Pro 6.0 (Molecular Devices).

### Array Data Normalization and Statistical Analysis

Data sets for rat and mouse EE and mouse TAM have been previously published [21,37] and have been integrated into the current comparative analysis with rat TAM data. As previously described with these data sets, rat TAM microarray data were first examined using a quality assurance protocol prior to further analysis to ensure consistent, high quality data throughout the dose-response and time course studies prior to normalization and further analysis [38]. Data were normalized using a semi-parametric approach [39]. Model-based t-values were calculated from normalized data, comparing treated from VEH responses per time-point. Empirical Bayes analysis was used to calculate posterior probabilities of activity (P1(*t*)-value) on a per gene and time-point basis using the model-based t-value [40]. Genes were filtered for differential expression based on the P1(*t*)-value which indicates increasing activity as the value approaches 1.0. A two-tiered set of criteria including a statistical P1(*t*) > 0.999 and |fold-change| > 1.5 was used as an initial selection filter of the expression data and defined initial differentially expressed gene lists in both the rat and mouse. All data was deposited into dbZach , a MIAME compliant relational database that ensures proper data management and facilitates data analysis. Complete data sets with annotation and P1(*t*) values are available in Additional Files 3, 4, 5, and 6.

### Expression Data Annotation and Coactivity Analysis

Features refer to unique cDNA clones spotted on the array and are assigned a GenBank accession number, gene name, symbol, and Entrez Gene ID where annotation is available. For the sake of brevity and consistency, genes are referenced by their official gene symbol as defined by NCBI. Rat and mouse orthologous gene pairs were derived from the publicly available Ensembl and HomoloGene databases. Comparing gene expression between species or ligand treatment involved examining the time, direction, and statistical significance of the change in expression on a gene by gene basis. Similarities and differences in gene expression patterns were designated as coactive-similar direction (CAS), coactive-divergent direction (CAD), displaced active-similar direction (DAS), and displaced active-divergent direction (DAD). Comparative analysis was conducted using a multivariate correlation-based visualization application developed in-house. Additional analysis was performed using the **4-way Venn Diagram Generator **(4VDG, , which involved applying an initial relaxed filtering criteria (|fold change| > 1.3 and P1(*t*) > 0.99) to each data set, then entering respective Entrez Gene IDs (mouse IDs were used for rat where HomoloGene indicated orthology) into the 4VDG. The output categories were then filtered for only those genes which met more stringent criteria (|fold change| > 1.5 and P1(*t*) > 0.999) at any one time point. Gene lists for each 4-way Venn category are available [see Additional File 7].

### QRT-PCR (Real Time) Analysis

For each sample, 1.0 μg of total RNA was reverse transcribed by SuperScript II using an anchored oligo-dT primer as described by the manufacturer (Invitrogen). The resultant cDNA (1.0 μl) was used as the template in a 30 μl PCR reaction containing 0.1 μM each of forward and reverse gene-specific primers designed using Primer3 (15), 3 mM MgCl_2_, 1.0 mM dNTPs, 0.025 IU AmpliTaq Gold and 1× SYBR Green PCR buffer (Applied Biosystems, Foster City, CA). Gene names, accession numbers, forward and reverse primer sequences and amplicon sizes are available [see Additional File 8]. PCR amplification was conducted in MicroAmp Optical 96-well reaction plates (Applied Biosystems) on an Applied Biosystems PRISM 7500 Sequence Detection System using the following conditions: initial denaturation and enzyme activation for 10 min at 95°C, followed by 40 cycles of 95°C for 15 s and 60°C for 1 min. A dissociation protocol was performed to assess the specificity of the primers and the uniformity of the PCR generated products. Each plate contained duplicate standards of purified PCR products of known template concentration covering six orders of magnitude to interpolate relative template concentrations of the samples from the standard curves of log copy number versus threshold cycle (Ct). No template controls (NTC) were also included on each plate. Samples with a Ct value within 2 SD of the mean Ct values for the NTCs were considered below the limits of detection. The copy number of each unknown sample for each gene was standardized to the geometric mean of house-keeping gene, Rpl7 to control for differences in RNA loading, quality and cDNA synthesis. Statistical significance of induced or repressed genes was determined using two-way ANOVA followed by t-test for VEH treatment comparisons (SAS 9.1, SAS Institute Inc.). For graphing purposes, the relative expression levels were scaled such that the expression level of the time-matched control group was equal to one.

## Authors' contributions

JK supervised and participated in all in-life studies, carried out gene expression experiments and analysis and drafted the manuscript. AF coordinated and participated in the in-life studies and histological processing. LB carried out statistical analysis and provided bioinformatics support in data management. KW carried out histopathological assessments and analysis. TZ conceived the study, participated in design of the study and participated in final draft of manuscript. All authors read and approved the final manuscript.

## Pre-publication history

The pre-publication history for this paper can be accessed here:



## Supplementary Material

Additional file 1 **Uterine Relative Wet Weights**. data presented represents averaged weights of unblotted whole rodent uteri Click here for file

Additional file 2 **Uterine Relative Water Content**. data presented represents average difference between wet and blotted uterine weight Click here for file

Additional file 3 **Mouse EE Gene Expression Data**. data presented represents averaged fold changes and statistical values per time point for each feature on our array platform for mouse ethynylestradiol. Click here for file

Additional file 4 **Mouse TAM Gene Expression Data**. data presented represents averaged fold changes and statistical values per time point for each feature on our array platform for mouse tamoxifen. Click here for file

Additional file 5 **Rat EE Gene Expression Data**. data presented represents averaged fold changes and statistical values per time point for each feature on our array platform for rat ethynylestradiol. Click here for file

Additional file 6 **Rat TAM Gene Expression Data**. data presented represents averaged fold changes and statistical values per time point for each feature on our array platform for rat tamoxifen Click here for file

Additional file 7 **4-Way Venn Analysis Gene Sets**. data presented represents gene lists for each sub group in the 4-way Venn analysis. Click here for file

Additional file 8 **QRT-PCR Primer Sequences**. data presented represents the DNA sequences of gene-specific primers used for quantitative real-time PCR analysis. Click here for file
